# The expression and role of protein kinase C (PKC) epsilon in clear cell renal cell carcinoma

**DOI:** 10.1186/1756-9966-30-88

**Published:** 2011-09-28

**Authors:** Bin Huang, Kaiyuan Cao, Xiubo Li, Shengjie Guo, Xiaopeng Mao, Zhu Wang, Jintao Zhuang, Jincheng Pan, Chengqiang Mo, Junxing Chen, Shaopeng Qiu

**Affiliations:** 1 Department of Urology, the First Affiliated Hospital, Sun Yat-Sen University, Guangzhou (510080), China; 2 Research Center for Clinical Laboratory Standard, Zhongshan Medical School, Sun Yat-sen University, Guangzhou (510080), China; 3 Pulmonary disease institute, Guangzhou Chest Hospital Pulmonary Disease Institute, Guangzhou (510095), China; 4 Department of Urology, Sun Yat-Sen University Cancer Center, Guangzhou (510060), China

**Keywords:** Protein kinase C epsilon, Renal cell carcinoma, Clear cell

## Abstract

Protein kinase C epsilon (PKCε), an oncogene overexpressed in several human cancers, is involved in cell proliferation, migration, invasion, and survival. However, its roles in clear cell renal cell carcinoma (RCC) are unclear. This study aimed to investigate the functions of PKCε in RCC, especially in clear cell RCC, to determine the possibility of using it as a therapeutic target. By immunohistochemistry, we found that the expression of PKCε was up-regulated in RCCs and was associated with tumor Fuhrman grade and T stage in clear cell RCCs. Clone formation, wound healing, and Borden assays showed that down-regulating PKCε by RNA interference resulted in inhibition of the growth, migration, and invasion of clear cell RCC cell line 769P and, more importantly, sensitized cells to chemotherapeutic drugs as indicated by enhanced activity of caspase-3 in PKCε siRNA-transfected cells. These results indicate that the overexpression of PKCε is associated with an aggressive phenotype of clear cell RCC and may be a potential therapeutic target for this disease.

## Background

Renal cell carcinoma (RCC) accounts for approximately 3% of all malignant tumors in adults, which afflicts about 58, 240 people and causes nearly 13, 040 deaths each year in USA [1]. RCCs are classified into five major subtypes: clear cell (the most important type, accounts for 82%), papillary, chromophobe, collecting duct, and unclassified RCC [2]. Operation is the first treatment choice for RCC; however, some patients already have metastasis at the time of diagnosis and are resistant to conventional chemotherapy, radiotherapy, and immunotherapy [3]. Thus, a more effective anti-tumor therapy is urgently needed.

Protein kinase C (PKC), a family of phospholipid-dependent serine/threonine kinases, plays an important role in intracellular signaling in cancer [4-8]. To date, at least 11 PKC family members have been identified. PKC isoenzymes can be categorized into three groups by their structural and biochemical properties: the conventional or classical ones (α, βI, βII, and γ) require Ca^2+ ^and diacylglycerol (DAG) for their activation; the novel ones (δ, ε, η, and θ) are dependent on DAG but not Ca^2+^; the atypical ones (ζ and λ/ι) are independent of both Ca^2+ ^and DAG [4-6]. Among them, PKCε is the only isoenzyme that has been considered as an oncogene which regulates cancer cell proliferation, migration, invasion, chemo-resistance, and differentiation via the cell signaling network by interacting with three major factors RhoA/C, Stat3, and Akt [9-13]. PKCε is overexpressed in many types of cancer, including bladder cancer [14], prostate cancer [15], breast cancer [16], head and neck squamous cell carcinoma [17], and lung cancer [18] as well as RCC cell lines [19,20]. The overexpression and functions of PKCε imply its potential as a therapeutic target of cancer.

In this study, we detected the expression of PKCε in 128 human primary RCC tissues and 15 normal tissues and found that PKCε expression was up-regulated in these tumors and correlated with tumor grade. Furthermore, PKCε regulated cell proliferation, colony formation, invasion, migration, and chemo-resistance of clear cell RCC cells. Those results suggest that PKCε is crucial for survival of clear cell RCC cells and may serve as a therapeutic target of RCC.

## Methods

### Samples

We collected 128 specimens of resected RCC and 15 specimens of pericancerous normal renal tissues from the First Affiliated Hospital of the Sun Yat-sen University (Guangzhou, China). All RCC patients were treated by radical nephrectomy or partial resection. Of the 128 RCC samples, 10 were papillary RCC, 10 were chromophobe RCC, and 108 were clear cell RCC according to the 2002 AJCC/UICC classification. The clear cell RCC samples were from 69 male patients and 39 female patients at a median age of 56.5 years (range, 30 to 81 years). Tumors were staged according to the 2002 TNM staging system [21] and graded according to the Fuhrman four-grade system [22]. Informed consent was obtained from all patients to allow the use of samples and clinical data for investigation. This study was approved by the Ethics Council of the Sun Yat-sen University for Approval of Research Involving Human Subjects.

### Cell culture

Five human RCC cell lines 769P, 786-O, OS-RC-2, SN12C, and SKRC39 were used in this research. Clear cell RCC cell lines 769P and 786-O were purchased from the American Type Culture Collection (Rockville, MD); RCC cell lines OS-RC-2, SN12C, and SKRC39 were a kind gift from Dr. Zhuowei Liu (Department of Urology, Sun Yat-sen University Cancer Center). 769P, 786-O, OS-RC-2, and SKRC39 cells were cultured in RPMI-1640 (Gibco, Carlsbad, California); SN12C cells were maintained in Dulbeccos's modified Eagle's medium (DMEM, Gibco) containing 10% fetal calf serum (FCS, Gibco, Carlsbad, California), 1% (v/v) penicillin, and 100 μg/ml streptomycin at 37°C in a 5% CO_2 _atmosphere.

### Immunohistochemistry and scoring for PKCε expression

All 5-μm thick paraffin sections of tissue samples were deparaffinized with xylene and rehydrated through graded alcohol washes, followed by antigen retrieval by heating sections in sodium citrate buffer (10 mM, pH 6.0) for 30 min. Endogenous peroxidase activity was blocked with 30 min incubation in methanol containing 0.03% H_2_O_2_. The slides were then incubated in PBS (pH 7.4) containing normal goat serum (dilution 1:10) and subsequently incubated with monoclonal mouse IgG1 anti-PKCε antibody (610085; BD Biosciences, BD, Franklin Lakes, NJ USA) with 1:200 dilution at 4°C overnight. Following this step, slides were treated with biotin-labeled anti-IgG and incubated with avidin-biotin peroxidase complex. Reaction products were visualized by diaminobenzidine (DAB) staining and Meyer's hematoxylin counterstaining. Negative controls were prepared by replacing the primary antibody with mouse IgG1 (I1904-79G, Stratech Scientific Ltd, UK). Phosphate-buffered saline instead of primary antibody was used for blank controls.

Three independent pathologists blinded to clinical data scored PKCε immunohistochemical staining of all sections according to staining intensity and the percentage of positive tumor cells as follows [23,24]: no staining scored 0; faint or moderate staining in ≤ 25% of tumor cells scored 1; moderate or strong staining in 25% to 50% of tumor cells scored 2; strong staining in ≥50% of tumor cells scored 3. For each section, 10 randomly selected areas were observed under high magnification and 100 tumor cells in each area were counted to calculate the proportion of positive cells. Overexpression of PKCε was defined as staining index ≥2. Immunohistochemical reactions for all samples were repeated at least three times and typical results were illustrated.

### Western blot analysis for PKCε expression

The expression of PKCε in 769P, 786-O, OS-RC-2, SN12C, and SKRC39 cells was detected by Western blot as described previously [25]. Briefly, total proteins were extracted from RCC cell lines and denatured in sodium dodecyl sulfate (SDS) sample buffer, then equally loaded onto 10% polyacrylamide gel. After electrophoresis, the proteins were transferred to a polyvinylidene difluoride membrane. Blots were incubated with the indicated primary antibodies overnight at 4°C and detected with horseradish peroxidase-conjugated secondary antibody. The monoclonal anti-PKCε antibody was used at the dilution of 1:3, 000, whereas anti-GAPDH (sc-137179; Santa Cruz Biotechnology, Santa Cruz, CA, USA) was used at the dilution of 1:2, 000.

### Immunocytochemistry for PKCε expression and location

769P cells were washed with 1× PBS and fixed in 4% paraformaldehyde for 10 min at room temperature, blocked in 0.1% PBS-Tween solution containing 5% donkey serum (*v/v*) at room temperature for 1 h, and incubated overnight with anti-PKCε antibody (1:300) in blocking solution. Then cells were washed three times for 10 min with 0.1% PBS-Tween and incubated for 1 h with secondary antibody in blocking solution. DyLight488-conjugated AffiniPure donkey anti-mouse IgG (H + L) was used at the dilution of 1:500 (715485151, Jackson ImmunoResearch Europe, Newmarket, Suffolk, UK). After incubation, cells were washed three times with 0.1% PBS-Tween, counterstained with Hoechst 33342, and mounted for confocal microscopy. The expression and location of PKCε in cells were observed under a fluorescent microscope.

### RNA interference (RNAi) to knockdown PKCε in 769P cells

As described in literature [26-28], 769P cells were transfected with small interfering RNA (siRNA) against PKCε (sc-36251) and negative control siRNA (sc-37007) by Lipofectamine 2000 transfection reagent and Opti-MEMTM (Invitrogen, Carlsbad, CA, USA) according to the manufacturer's protocol. All siRNAs were obtained from Santa Cruz Biotechnology. Briefly, 1 × 10^5 ^769P cells were plated in each well of 6-well plates and cultured to reach a 90% confluence. Cells were then transfected with siRNA by using the transfection reagent in serum-free medium. Total cellular proteins were isolated at 48 h after transfection. PKCε expression was monitored by reverse transcription-polymerase chain reaction (RT-PCR) and Western blot using the anti-PKCε antibody mentioned above.

### Reverse transcription-polymerase chain reaction

Total RNA was isolated from 769P cells transfected with PKCε siRNA or control siRNA, or from untransfected cells using TRIzol Reagent (Invitrogen) as per the manufacturer's protocol, and subjected to reverse transcription using reverse transcriptase Premix Ex Taq (Takara, Otsu, Japan). The sequences of PKCε primers used for PCR were as follows: forward, 5'-ATGGTAGTGTTCAATGGCCTTCT-3'; reverse, 5'-TCAGGGCATCAGGTCTTCAC-3'. The sequences of internal control glyceraldehyde-3-phosphate dehydrogenase (GAPDH) were as follows: forward, 5'-ATGTCGTGGAGTCTACTGGC-3'; reverse, 5'-TGACCTTGCCCACAGCCTTG-3'. PKCε was amplified by 30 cycles of denaturation at 95°C for 1 min, annealing at 60°C for 30 s, extension at 72°C for 2 min, and final extension at 72°C for 8 min. The products were resolved on a 1% agarose gel containing ethidium bromide for electropheresis.

### Colony formation assay

Cell proliferation was assessed by colony formation assay. PKCε siRNA-transfected, control siRNA-transfected, and untransfected 769P cells were seeded in a 6-well plate (1 × 10^3 ^cells/well), and cultured in complete medium for 1 week. Cell colonies were then visualized by 0.25% crystal violet. After washing out the dye, colonies containing > 50 cells were counted. The colony formation efficiency (CFE) was the ratio of the colony number to the planted cell number.

### Wound-healing assay

Cell migration was evaluated by a scratched wound-healing assay on plastic plate wells. In brief, 769P cells were seeded in a 6-well plate (5 × 10^5 ^cells/well) and grew to confluence. The monolayer culture was scratched with a sterile micropipette tip to create a denuded zone (gap) of constant width and the cell debris with PBS was removed. The initial gap length and the residual gap length at 6, 12, or 24 h after wounding were observed under an inverted microscope (ZEISS AXIO OBSERVER Z1) and photographed. The wound area was measured by the program Image J http://rsb.info.nih.gov/ij/. The percentage of wound closure was estimated by 1 - (wound area at Tt/wound area at T0) × 100%, where Tt is the time after wounding and T0 is the time immediately after wounding.

### Invasion assay

Cell invasion was assessed using the CHEMICON cell invasion assay kit (Millipore, Billerica, MA, USA) according to the manufacturer's instructions. In brief, 300 μl of warm serum-free medium was added into the interior of each insert (8 μm pore size) to rehydrate the extracellular matrix (ECM) layer for 2 h at room temperature, then it was replaced with 300 μl of prepared serum-free suspension of untransfected 769P cells, or cells transfected with PKCε siRNA or control siRNA (5 × 10^5 ^cells/ml); 500 μl of medium containing 10% fetal bovine serum was added to the lower chamber of the insert. Cells were incubated at 37°C in a 5% CO_2 _atmosphere for 24 h. After then, non-invading cells in the interior of the inserts were gently removed with a cotton-tipped swab; invasive cells on the lower surface of the inserts were stained with the staining solution for 20 min and counted under a microscope. All experiments were performed in triplicate.

### Drug sensitivity assay

At 48 h after siRNA transfection, transfected and untransfected cells were seeded into a 96-well plate at a density of 5 × 10^3 ^cells/well. After 24 h, cells were treated with various doses of sunitinib or 5-fluorouracil (Sigma, St Louis, MO, USA) for additional 48 h. Cell viability was measured by the MTT assay following the manufacturer's instructions. All experiments were performed in triplicate.

### Caspase-3 activity assay

The activity of caspase-3 was determined using the caspase-3 activity kit (Beyotime, Haimen, China), based on the ability of caspase-3 to change acetyl-Asp-Glu-Val-Asp p-nitroanilide (Ac-DEVD-pNA) into a yellow formazan product p-nitroaniline (pNA) [29,30]. According to the manufacturer's protocol, cell lysates of transfected and untransfected 769P cells after drug treatment as described above were centrifuged at 12, 000 × *g *for 15 min at 4°C, and protein concentrations were determined by Bradford protein assay. Cellular extracts (30 μg) were incubated in a 96-well microtitre plate with 10 μl Ac-DEVD-pNA (2 mM) for 6 h at 37°C. Then caspase-3 activity was quantified in the samples with a microplate spectrophotometer (NanoDrop 2000c, Thermo Fisher Scientific Inc., USA) by the absorbance at a wavelength of 405 nm. All experiments were performed in triplicate.

### Statistical analysis

Statistical analysis was performed using the SPSS 13.0 software. The relationship between PKCε expression and the clinicopathologic features of RCC was assessed by the Fischer's exact test. Continuous data are expressed as mean ± standard deviation. Statistical significance was analyzed by one-way analysis of variance (ANOVA) followed by Bonferroni's post-hoc test, with values of *P *< 0.05 considered statistically significant.

## Results

### PKCε expression in renal tissues

**Figure 1:**
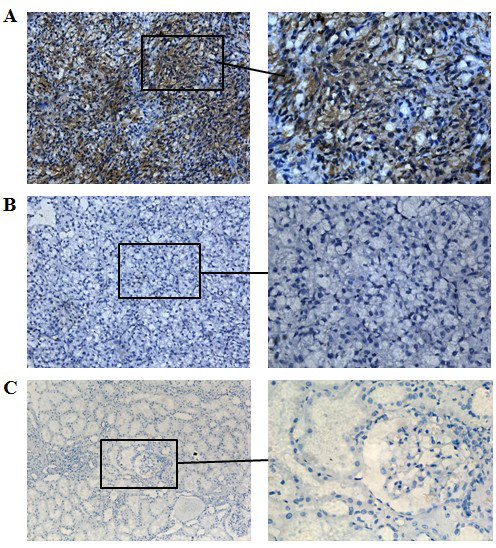
**Immunohistochemical staining of PKCε in tissue specimens**. PKCε is overexpressed in both cytoplasm and nuclei of clear cell renal cell carcinoma (RCC) cells **(A)**. Primary antibody isotype control **(B) **and normal renal cells **(C) **show no or minimal staining. The original magnification was ×200 for left panels and ×400 for right panels.

The expression of PKCε protein in 15 specimens of normal renal tissues and 128 specimens of RCC was detected by immunohistochemistry with an anti-PKCε monoclonal antibody. PKCε expression was weak in normal renal tissues, but strong in both cytoplasm and nuclei of RCC cells (Figure 1). The level of PKCε overexpression was significantly higher in RCC than in normal tissues (63.3% vs. 26.7%, *P *= 0.006). When stratified by pathologic type, no significant difference was observed among clear cell, papillary, and chromophobe RCCs (62.0% vs. 60.0% and 80.0%, *P *= 0.517). PKCε overexpression showed no relationship with the sex and age of patients with clear cell RCC (both *P *> 0.05), but was related with higher T stage (*P *< 0.05) and higher Fuhrman grade (*P *< 0.01) (Table 1).

**Table 1:** PKCε overexpression in human clear cell renal cell carcinoma tissues

Group	Cases	PKCε overexpression		*P *value
			
		(-)	(+)	
**Sex**				
Men	69	24	45	0.365
Women	39	17	22	
**Age**				
≤ 55 years	43	16	27	0.599
>55 years	65	21	44	
**T stage**				
T_1/_T_2_	89	38	51	0.028
T_3_/T_4_	19	3	16	
**Fuhrman grade**				
G_1_/G_2_	86	39	47	0.002
G_3_/G_4_	22	2	20	

PKCε, protein kinase C epsilon.

### PKCε expression in renal cell cancer cell lines

**Figure 2:**
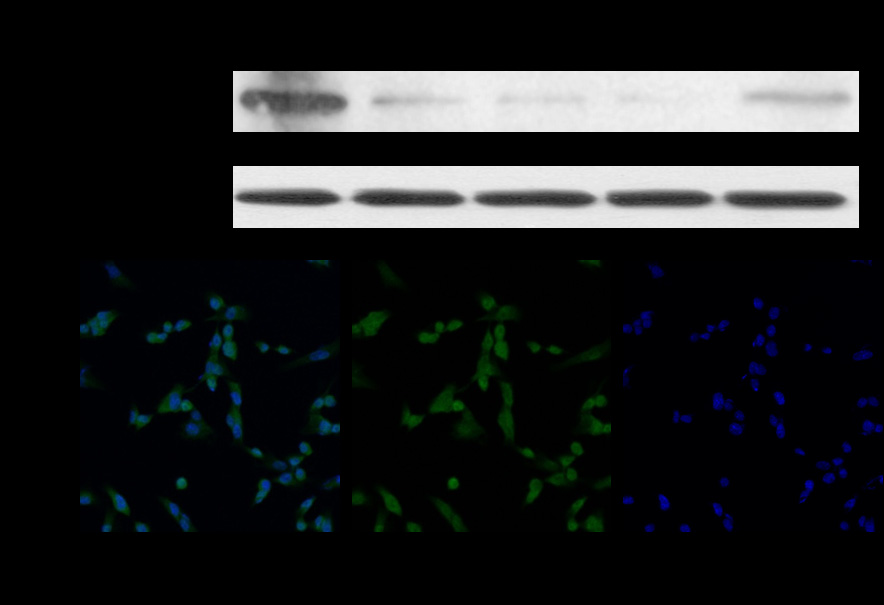
**Expression of PKCε in renal cell carcinoma (RCC) cell lines**. **A**. Western blot shows that PKCε is expressed in all five RCC cell lines, with the highest level in 769P cells. GAPDH is the loading control. **B**. Immunocytochemical staining with PKCε antibody shows that PKCε is mainly expressed in cytoplasm and nuclei of 769P cells (original magnification×200). Green fluorescence indicates PKCε-positive cells, whereas blue fluorescence indicates the nuclei of the cells. The first panel is a merge image of the latter two.

We detected the expression of PKCε in five RCC cell lines using Western blot. PKCε was expressed in all five RCC cell lines at various levels, with the maximum level in clear cell RCC cell line 769P (Figure 2A). Immunocytochemical staining showed that PKCε was mainly expressed in both cytoplasm and nuclei, sometimes on the membrane, of 769P cells (Figure 2B).

### Effects of PKCε on proliferation, migration, and invasion of 769P cells

**Figure 3:**
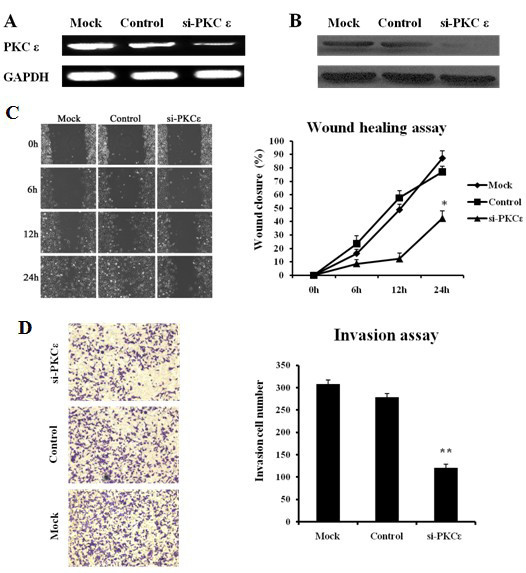
**Effects of PKCε knockdown on migration, and invasion of 769P cells**. 769P cells were transfected with PKCε small interfering RNA (siRNA) or control siRNA; untransfected cells were used as blank control. GAPDH was used as internal control. Both reverse transcription-polymerase chain reaction **(A) **and Western blot **(B) **show that PKCε expression is inhibited after PKCε RNAi. **C**. The wound-healing assay shows a significant decrease in the wound healing rate of 769P cells after PKCε siRNA transfection (*, *P *< 0.05). **D**. Invasion assay shows a significant decrease in invaded 769P cells after PKCε siRNA transfection (**, *P *< 0.01).

To examine the functions of PKCε, we knocked down PKCε by transfecting PKCε siRNA into 769P cells. The mRNA and protein expression of PKCε was significantly weaker in PKCε siRNA-transfected cells than in control siRNA-transfected cells and untransfected cells (Figure 3A and 3B). The colony formation assay revealed that cell colony formation efficiency were lower in PKCε siRNA-transfected cells than in control siRNA-transfected and untransfected cells [(29.6 ± 1.4)% vs. (60.9 ± 1.5)% and (50.9 ± 1.1)%, *P *< 0.05], suggesting that PKCε may be important for the growth and survival of RCC cells.

The wound-healing assay also demonstrated significant cell migration inhibition in PKCε siRNA-transfected cells compared with control siRNA-transfected and untransfected cells at 24 h after wounding [wound closure ratio: (42.6 ± 5.3)% vs. (77.1 ± 4.1)% and (87.2 ± 5.5)%, *P *< 0.05] (Figure 3C). The CHEMICON cell invasion assay demonstrated that the number of invading cells was significantly decreased in PKCε siRNA group compared with control siRNA and blank control groups (120.9 ± 8.1 vs. 279.0 ± 8.3 and 308.5 ± 8.8, *P *< 0.01) (Figure 3D). Our data implied that PKCε knockdown also inhibited cell migration and invasion *in vitro*.

### Knockdown of PKCε sensitizes 769P cells to chemotherapy *in vitro*

**Figure 4:**
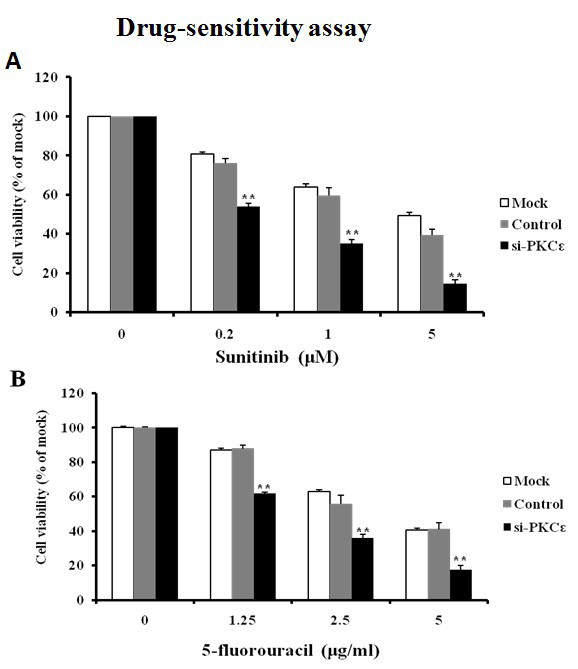
**Knockdown of PKCε sensitizes 769P cells to Sunitinib (A) and 5-fluorouracil (B)**. 769P cells were transfected with PKCε siRNA or control siRNA; untransfected cells were used as blank control. At 72 h after siRNA transfection, cells were treated with sunitinib (0.2, 1, and 5 μM) or 5-fluorouracil (1.25, 2.5, and 5 μg/ml) for another 48 h. MTT assay shows increased sensitivity of cells to sunitinib and 5-fluorouracil after siRNA transfection (**, *P *< 0.01).

As PKCε is involved in drug resistance in some types of cancer and adjuvant chemotherapy is commonly used to treat RCC, we tested whether PKCε is also involved in drug response of RCC cell lines. Both siRNA-transfected and untransfected 769P cells were treated with either sunitinib or 5-fluorouracil. The survival rates of 769P cells after treatment with Sunitinib and 5-fluorouracil were significantly lower in PKCε siRNA group than in control siRNA and blank control groups (all *P *< 0.01) (Figure 4).

**Figure 5:**
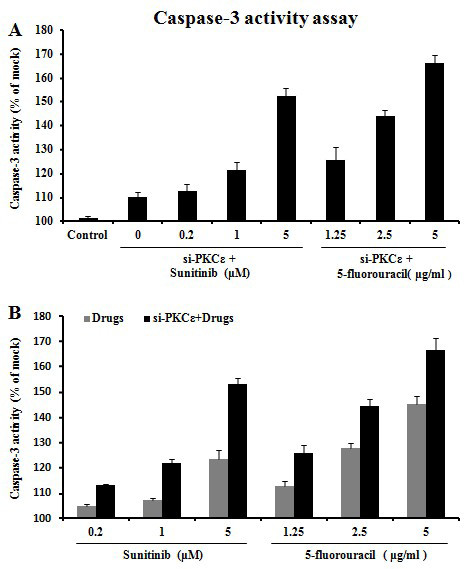
**Changes of caspase-3 activity in 769P cells after PKCε downregulated and cytotoxic drug treatment**. 769P cells were transfected with PKCε siRNA; untransfected cells were used as blank control. At 72 h after siRNA transfection, cells were treated with indicated doses of sunitinib or 5-fluorouracil. Panel A shows that the caspase-3 activity was significantly higher in PKCε siRNA-transfected cells, either with or without drug treatment, than in untransfected cells (*P *< 0.01) and was higher in the cells underwent both siRNA transfection and drug treatment than in those underwent only siRNA transfection (*P *< 0.05). Panel B shows that the caspase-3 activity was significantly higher in the cells underwent both siRNA transfection and drug treatment than in those underwent only drug treatment (*P *< 0.05).

Caspase-3 is the final executor of apoptotic DNA damage, and its activity is a characteristic of apoptosis [10]. We next examined cell apoptosis after siRNA transfection and treatment with cytotoxic drug sunitinib or 5-fluorouracil. At 48 h, the caspase-3 activity was significantly higher in PKCε siRNA-transfected cells, either with or without drug treatment, than in untransfected cells (*P *< 0.01) (Figure 5A), and was significantly higher in the cells underwent both siRNA transfection and drug treatment than in those underwent only drug treatment (*P *< 0.05) (Figure 5B), suggesting that PKCε may contribute to the resistance of clear cell RCC cells to cytotoxic drugs.

## Discussion

Increasing evidences indicate that PKCε is overexpressed in various tumor tissues and functions as a transforming oncogene [14-20]. To explore the oncogenic potential of PKCε, Mischak et al. [31] overexpressed PKCε in NIH 3T3 fibroblasts and observed accelerated growth of cells with PKCε overexpression. In addition, tumors were developed in all mice injected with PKCε-overexpressing NIH 3T3 cells. In the same year, Cacace et al. [32] confirmed the oncogenic role of PKCε in fibroblasts. Similarly, Perletti *et al*. [33] found that PKCε overexpression in colonic epithelial cells led to a metastatic phenotype, including morphological changes, increased anchorage-independent growth and tumorigenesis in a xenograft model. We also found that PKCε was overexpressed in RCC tissues as compared with that in normal renal tissues and that PKCε was closely related to higher grades of clear cell RCC. PKCε was also expressed in all five human RCC cell lines used in our study.

PKCε has been shown to regulate many cellular processes, including cell proliferation, migration, invasion, chemo-resistance, apoptosis, and differentiation [9-12]. Multiple mechanisms are involved in PKCε-regulated tumorigenesis. For example, PKCε promotes cell proliferation and survival by regulating the Ras signaling pathway, which is a well characterized signaling pathway in cancer biology [10,34]. PKCε expression is related to the activation of cyclin D1 promoter, a downstream effects of Ras signaling, and to enhanced cell growth [9-11]. In addition, PKCε plays a role in anti-apoptotic signaling pathways through interacting with caspases and Bcl-2 family members [35,36], and exerts its pro-survival effects by activating Akt/PKB [27,37]. These mechanisms may explain the inhibited growth of RCC cells by PKCε knockdown in our study.

Like in other cancer types, relapse and metastasis are the main causes of failure of surgical operation in treating clear cell RCC. Patients with RCC response to postoperative adjuvant chemotherapy at various levels and usually cannot achieve expected outcomes [3]. The phenotype of tumor metastasis presents with promotion of cell proliferation, escape from apoptosis, and dysregulation of cellular adhesion and migration. The invasion of tumor cells to surrounding tissues and spreading to distal sites rely on cell migration ability. Cell migration, a complex event, depends on the coordinated remodeling of the actin cytoskeleton, regulated assembly, and turnover of focal adhesion [11]. Interestingly, PKCε contains an actin-binding domain [12] and promotes F-actin assembly in a cell-free system, indicating that PKCε modulates cell migration via actin polymers. In addition, PKCε has been observed to translocate to the cell membrane during the formation of focal adhesions [38] and to reverse the effect of non-signaling β1-integrin molecules in inhibiting cell spreading [39]. PKCε-driven cell migration was shown to be mediated, at least in part, by activating downstream small Rho GTPases, especially RhoA and/or RhoC [17]. We found that silencing PKCε by RNAi decreased migration and invasion of clear cell RCC cells *in vitro*, suggesting that PKCε may be one of the potential treatment targets for this disease. Additionally, PKCε is also cleaved by caspases in response to several apoptotic stimuli including chemotherapeutic agents. PKCε is a substrate for caspase-3 as evidenced by caspase-3-caused PKCε cleavage and the inhibition of PKCε cleavage by a cell permeable inhibitor of caspase-3 [40]. PKCε has been shown to regulate apoptosis mediated by either DNA damage or receptor [10]. PKCε up-regulation was associated with chemoresistance of non-small cell lung cancer (NSCLC) cell lines, whereas chemosensitivity was proved in PKCε-knockdown SCLC cells [41]. In addition, PKCε was reported to mediate with induction of the drug-resistance gene P-glycoprotein in LNCaP cells [42]. In our study, PKCε knockdown enhanced the activity of pro-apoptotic gene caspase-3 and sensitized 769P cells to chemotherapy, indicating the association between PKCε and chemosensitivity of RCC.

## Conclusions

Our results confirm the role of PKCε as an oncogene in RCC, especially in the subtype of clear cell, suggesting that PKCε might be a potential treatment target for this disease, which warrants verification in further studies.

## Competing interests

The authors declare that they have no competing interests.

## Authors' contributions

JTZ, JCP and CQM evaluated the immunostainings. BH have made substantial contributions to acquisition of data. XBL, SJG and ZW performed the statistical analysis. BH, JXC and SPQ participated in the design of the study. BH and KYC drafted the manuscript. XPM and SPQ revised the manuscript. All authors read and approved the final manuscript.
